# Bio-accumulation of Arsenic (III) Using Nelumbo Nucifera Gaertn

**DOI:** 10.5696/2156-9614-9.23.190902

**Published:** 2019-07-23

**Authors:** Archana Saily Painuly, Ruchi Gupta, Sidharth Vats

**Affiliations:** Shri Ramswaroop Memorial University, Faculty of Chemical Sciences, Uttar Pradesh, India

**Keywords:** arsenic, biosorption, biomass, *Nelumbo nucifera* Gaertn (Lotus)

## Abstract

**Background.:**

High arsenic levels in potable water are a threat to public health in India. About 85% of the water in India's rural areas comes from groundwater and roughly 27 million people are at risk of arsenic (As) contamination.

**Objectives.:**

The present study was performed to examine the feasibility of providing an effective and affordable means for arsenic abatement in socio-economically poor and rural areas in India. This is the first report on the effectiveness of powder Nelumbo nucifera Gaertn (lotus) root biomass for As (III) eradication from aqueous solution.

**Methods.:**

Batch experiments were conducted to determine the effects of various operating parameters, including pH, initial As (III) ion concentration, adsorbent dosages, and contact time for As (III) sorption onto lotus root.

**Discussion.:**

The sorption efficiency of lotus root biomass for As (III) at pH 7 was found to be quantitative (96%) from 50 mg/L aqueous solution at a dose of 5gL^−1^. Capacity of the biosorbent for As (III) ion adsorption and the interaction between adsorbate with biosorbents were studied using Langmuir and Freundlich isotherm models. In the present study, the equilibrium parameter values ranged between 0 and 1, indicating that the adsorption of the As (III) ion onto lotus root biomass was favorable.

**Conclusions.:**

Lotus root powder biomass was found to be an effective adsorbent for As (III) and could be used as an efficient, cost-effective and environmentally safe biosorbent for the sorption of arsenic from aqueous solutions.

**Competing Interests.:**

The authors declare no competing financial interests.

## Introduction

Arsenic is a poison that has been identified as one of the world's greatest environmental hazards, threatening the lives of several hundred million people worldwide. Among all of the 105 arsenic-affected countries, the largest at-risk population is in Bangladesh, followed by West Bengal in India.^1^–^3^

**Figure 1:**
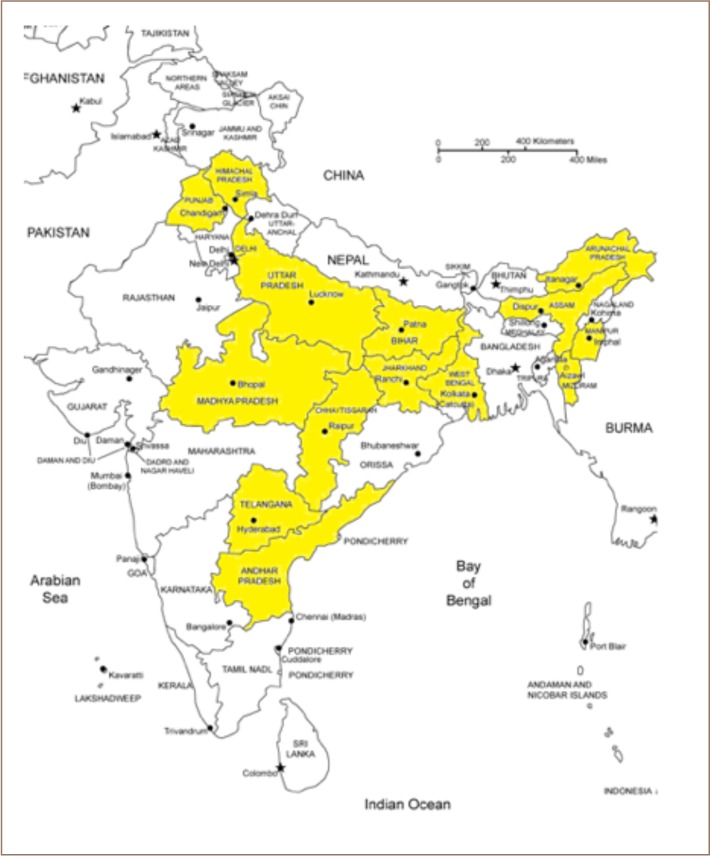
Arsenic affected areas

The first case of arsenic poisoning was diagnosed in West Bengal in 1983. Subsequently, the presence of arsenic in groundwater has been recorded in the central and eastern Indian Gangetic river plains, the northeastern states, and several other regions in India.^4^ The discovery of new sites every year as documented in a technical report published by the Central Ground Water Board of India has brought attention to the arsenic contamination situation among the scientific community as well as the public.^5^ The concentration of arsenic has risen above the concentration limits set for groundwater (range 1–4200 ppb) and surface water (range 0.3–300 ppb) in many states including Punjab, Jharkhand, Manipur, Mizoram, Arunachal Pradesh, Andhra Pradesh, Assam, Himachal Pradesh, Telangana Chhattisgarh, Uttar Pradesh, Bihar, and West Bengal in India (*Figure 1*).^4^,^6^–^8^ The unparalleled, unplanned and uncontrolled growth in medium-, small- and micro-level industries over the last 20 years has contributed to this situation. The low technical proficiency of entrepreneurs, outdated technology, and lower levels of spending on pollution control compared to large scale industry have exacerbated the problem. The adsorption method is known for its efficiency, low cost and eco-friendliness and provides an effective and affordable means for arsenic abatement for the population in low socioeconomic status and rural areas. The present study was performed to examine the feasibility of providing an effective and affordable means for arsenic abatement in socioeconomically poor and rural areas in India.

This is the first study on arsenic absorption and extraction using lotus (Nelumbo nucifera Gaertn) root biomass (lotus root biomass) according to a survey of the current literature.

## Methods

All chemicals used in the present study were of analytical reagent grade. De-ionized water was used in all of the experiments. A stock solution of As (III) was prepared from sodium meta-arsenite. The standard and test solutions were prepared diluting this solution with de-ionized water. One (1) M hydrochloric acid/1 M sodium hydroxide was used to obtain different pH values for the different experiments.

Batch experiments were conducted to investigate the effects of variables such as contact time, pH, initial As (III) concentration and lotus root biomass dose on As (III) sorption. Langmuir and Freundlich isotherm models were used to correlate the experimental equilibrium data.

### Preparation of biosorbent samples

Lotus roots as working substrate were collected from the local market of Sindholi, a small town near the city of Lucknow, Uttar Pradesh, India. Roots were cleaned with deionized water to remove soluble lighter materials. Cleaned roots were then cut into small pieces and air dried at room temperature for 5–6 days. The dried lotus root biomass was ground at 12000 rpm in a commercial blender using a stainless steel jar in a well ventilated area and then sieved to the desired particle size of 250–350 μm (−44+ 52 mesh size). Lotus root biomass was kept in air-tight containers for further use.

Phytochemical screening of the lotus root biomass revealed the presences of many biologically active compounds such as carbohydrates, reducing sugars, glycosides, proteins, steroids, phenols, flavones, tannins, saponins, alkaloids, anthroquinones, and quinines.^9^–^12^

**Abbreviations**
*RL*: Equilibrium parameter

Due to the presence of some possible functional groups known to exist in Nelumbo nucifera Gaertn, lotus root biomass can be used for sorption of heavy metals, cations, anions and dyes from aqueous solutions.^12^,^13^

## Batch bio-sorption studies

To carry out the experiment, a number of standard As (III) batch solutions (mg/L) were prepared from the stock As (III) solution. For each of the batch experiments, a known concentration of 50 ml As (III) was taken in a 250 ml conical flask with lotus root biomass with a range of concentrations (0.4% to 3.2% wt/vol). The mixture was shaken for a specific period of time (0–150 minutes) on a magnetic stirrer at room temperature and then filtered by Whatman grade 41 filter paper for analysis.

The effect of different operating conditions such as solution pH, contact time, agitation speed, As (III) ion concentration, and lotus root biomass dose was investigated by varying one of the operating conditions at a time, while the others were maintained constant. The residual concentration of As (III) was measured by taking out the samples from the test solution after definite time intervals until equilibrium was reached.

## Analysis of As (III) ions

The absorbance of the standard As (III) solutions was measured in a ultraviolet spectrophotometer at 524 nm, using safranine O as the coloring and complexing agent and the calibration curve was prepared.^14^ The absorbance of the test solutions was measured with the help of a calibration curve.^15^

## Calculation of arsenic sorption

The percentage sorption of arsenic ions from solutions was calculated as per Equation 1.^16^

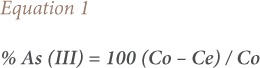



The adsorptive uptake of As (III) by lotus root was calculated according to Equation 2:

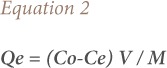
where, Qe is the As (III) concentration on the adsorbent at equilibrium, C_0_ is the initial As (III) concentration (mg/l), Ce is the aqueous phase As (III) concentration (mg/l) at equilibrium, V is the volume of the solution (l), and M is the mass of the adsorbent (g). Finally, the adsorption capacity Qe was plotted against the equilibrium concentration Ce. Each of the studies was conducted in triplicate, and the mean value (values ranges within ±2) was used for calculations.

## Sorption isotherms

Analysis of isotherm data was interpreted by using different isotherm models to determine the capacity of the biosorbent for metal ions and to describe how adsorbate interacts with biosorbents. In the present study, Langmuir and Freundlich models were tested. The Langmuir isotherm, which is the most commonly used model in biosorption, assumes that all sites have the same affinity and the secondary effects between sorbed species are negligible. The Langmuir equation is given by Equation 3:

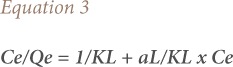
where, K_L_ (Lg^−1^) and a_L_ (Lmmol^−1^) is the Langmuir isotherm constant. A plot of Ce/Qe versus Ce gives a straight line of slope (a_L_/K_L_) and intercepts (1/K_L_), where K_L_/a_L_ gives the theoretical monolayer saturation capacity, Q_0_ (mg/g).

The Freundlich isotherm is an exponential equation and therefore assumes that the concentration of adsorbent surface enhances with the adsorbate concentration. Equation 4 is commonly applied in a heterogeneous system and is calculated as follows:

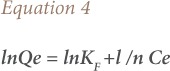
where, Qe is the solid phase sorbate concentration in equilibrium (mmol·g^−1^), *C*e is the liquid phase sorbate concentration in equilibrium (mmol·L^−1^), *K*_F_ is Freundlich constant (L·mg^1/n−1^·g^−1^), 1/n is the heterogeneity factor, and ln is the natural logarithms. Therefore, a plot of ln Qe versus ln *C*e enables the constant *K*_F_ and exponent 1/n to be determined.

## Results

**Figure 2:**
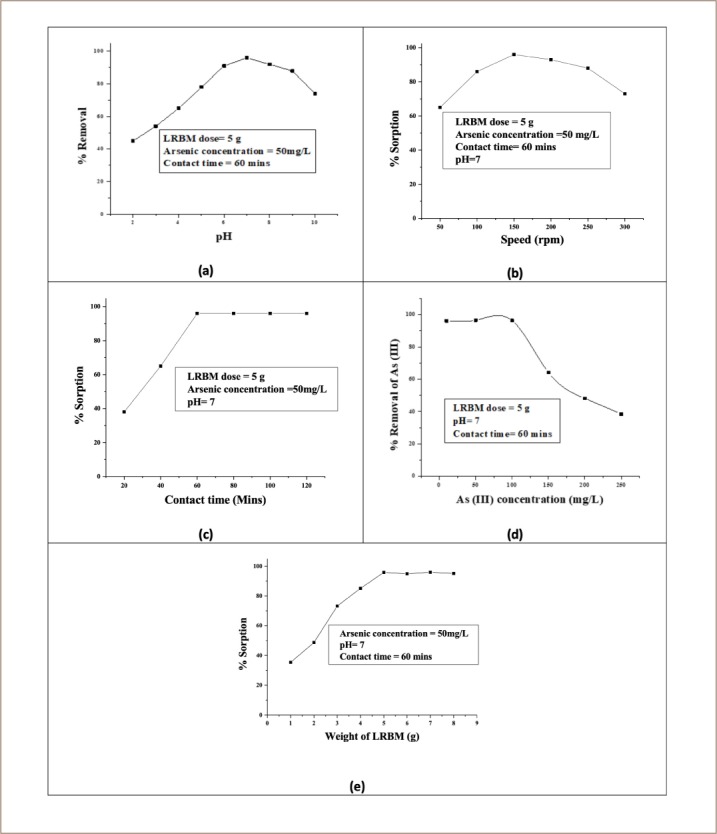
Effect of (a) pH (b) agitation (c) contact time (d) As (III) concentration and (e) bio-sorbent dose on arsenic bio-sorption by lotus root biomass

The effect of pH on the bio-sorption efficiency of lotus root biomass for arsenic sorption is shown in Figure 2a.

### Effect of agitation speed

The effect of varying agitation speeds (50–300 rpm) on the sorption of arsenic (III) was studied by taking constant arsenic concentrations of 50 mg/L, lotus root biomass 5 g, pH 7 for 1 hour at room temperature (*Figure 2b*).

### Effect of contact time

The effect of contact time on sorption of As (III) ion onto the lotus root biomass surface was investigated as shown in Figure 2c.

### Effect of arsenic concentration

Experiments were conducted by varying As (III) concentrations from 10–250 mg/L, 5 g lotus root biomass, at pH 7. Results are shown in Figure 2d.

### Effect of bio-sorbent dose

The effect of varying adsorbent dose (1–8 g) on sorption of As (III) was studied at pH 7, contact time of 60 minutes at room temperature and is represented in Figure 2e. As expected, the sorption of arsenic (III) increased from 35.4% to 95.8% with an increase in adsorbent dose until the dose was 5 g and subsequently remained almost constant.

### Equilibrium studies

**Figure 3:**
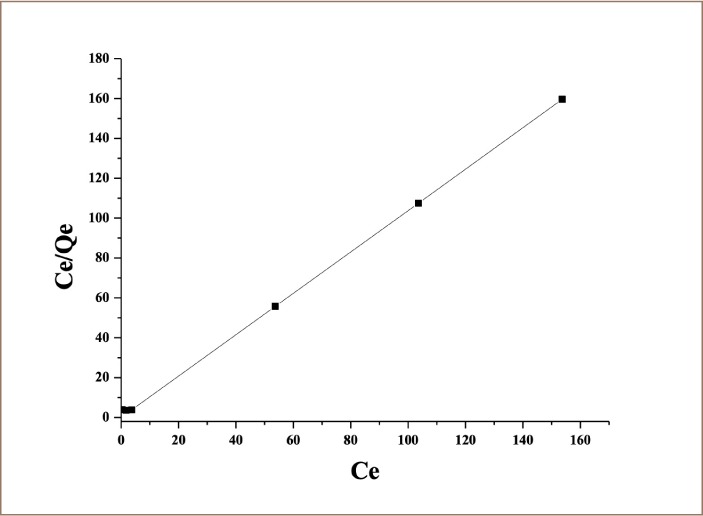
Langmuir adsorption isotherm [pH = 7, As (III) = 100 mg/L, lotus root biomass dose 5 g]

**Figure 4:**
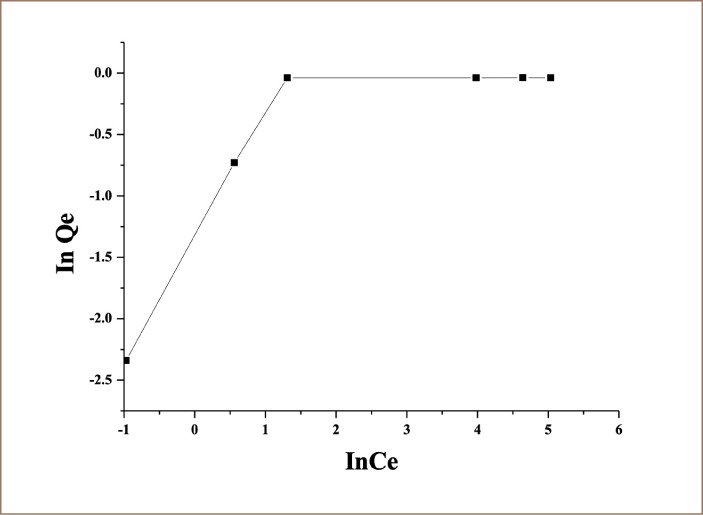
Freundlich adsorption isotherm [pH = 7, As (III) = 100 mg/L, lotus root biomass dose 5g]

Figure 3 and Figure 4 show Langmuir and Freundlich plots, respectively, for the adsorption of As (III) onto lotus root biomass, and Langmuir and Freundlich constants obtained from these plots are presented in Table 1. The results revealed that the Langmuir adsorption isotherm is the best model for the As (III) adsorption onto lotus root biomass with coefficient of determination of 0.99. The essential features of the Langmuir adsorption isotherm parameter can be used to predict the affinity between the sorbate and sorbent using a dimensionless constant called the separation factor or equilibrium parameter (*R*_*L*_), which is expressed by the following equation.^17^–^19^

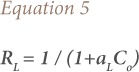
where, a_L_ is the Langmuir constant and C_o_ is the initial concentration.

**Table 1:** Comparison of Langmuir and Freundlich Isotherm Parameters and Calculated Theoretical Monolayer Saturation Capacity and Experimental As (III) Concentration on the Adsorbent at Equilibrium Value for Adsorption of Lotus Root Biomass

**Adsorbent**	**Langmuir isotherm**	**R^2^**	**a_L_/K_L_**	**1/K_L_**	**a_L_**	**R_L_**	**Q_e_**	**Q_0_**
lotus root biomass		0.99	1.04	3.901	0.265	0.04	0.964	0.963
**Adsorbent**	**Freundlich isotherm**	**R^2^**	**ln K_F_**	**1/n**	**K_F_**	**n**	**Q_e_**	**Q_0_**
lotus root biomass		0.79	0.29	−1.26	−1.23	0.793	0.963	0.488

Abbreviations: R^2^, coefficient of determination; a_L_/K_L_, slope; 1/K_L_, intercept; a_L_, Langmuir constant; Qe, As (III) concentration on the adsorbent at equilibrium; Q_0_, theoretical monolayer saturation capacity; ln, natural logarithms; K_F_, Freundlich constant; 1/n, heterogeneity factor; n, constant for a given adsorbate and adsorbent at a particular temperature

## Discussion

Surface properties of the bio-sorbent and the ionic form of the metal ion in solution are pH dependent.^20^–^22^ The pH can strongly influence the solution chemistry of the sorbates, the activity of functional groups on the biomass cell walls, as well as the competition of sorbates for the binding sites.^23^,^24^ Metal bio-sorption is not only dependent on pH, but also on one kind of biomass to the other.^25^ There was an insignificant change in the adjusted pH of the solution after addition of the adsorbent. The percentage sorption increased along with the pH of the aqueous phase and reached an optimum value of 96% sorption at pH 7. A similar trend has been reported in many previous studies.^16^,^26^–^29^ Adsorption capacity is highly dependent on the pH of the solution. Arsenic (III) exists primarily in the natural form of arsenous acid in the range of 6–8 pH where higher adsorption capacities were found. At a more alkaline pH, anionic As (III) species (Arsenous acid (H_2_AsO_3_^−^ and HAsO_3_^2−^)) predominate and sorption of arsenic decreases, possibly due to competition for active sites by excessive amounts of hydroxyl ions present in the water in an alkaline condition.^30^–^32^ The optimum pH was found to be 7 and this level was used for all further experiments.

As agitation speed increased, sorption capacity of lotus root biomass also increased up to 150 rpm from 65% to 96%. After 150 rpm, sorption capacity decreases and this may be attributed to the vortex formation, as possible cell disruption impairs sorption, leading to lower uptake of the metal ions.^33^ An agitation speed of 150 rpm was fixed for further experiments.

Arsenic sorption increased with increasing contact time up to 60 minutes. After 60 minutes, there was no change in sorption percentage, so 60 minutes was considered to be the effective equilibrium time for sorption. Initially the rate of sorption was higher, which may be due to more vacant sites for adsorption in the adsorbent, leading to a high solute concentration gradient.^32^

A decline in arsenic adsorption was observed after 100 mg/L. A decrease in As adsorption with concentration was attributed to dominance of repulsive forces towards the adsorbent at higher concentrations.

The percent As (III) sorption increased as lotus root biomass dosage increased due to the increase in surface area and availability of more active sites.^34^,^35^ However, in the present investigation, with further increases in adsorbent dose, the sorption extent remained almost constant, indicating the saturation of adsorption sites. The saturation of the active sites may also be due to the overlapping of active sites at a higher dosage, as well as a decrease in the effective surface area, resulting in the accumulation of exchanger particles.^32^ Therefore, 5 g was considered to be the optimum dose and was used in the present study.

The value of *R*_*L*_ indicated the type of Langmuir isotherm to be irreversible (*R*_*L*_ = 0), linear (*R*_*L*_ = 1), unfavorable (*R*_*L*_> 1), or favorable (0 < *R*_*L*_< 1).^36^ In the current study, *R*_*L*_ values ranged between 0 and 1, indicating that the adsorption of As (III) onto the lotus root biomass was favorable. The Freundlich plot for the entire concentration range is shown in Figure 4, exhibiting deviation from linearity. The plot can be divided into two regions, region 1 and region 2. It was evident that at the low concentration, data prior to saturation was well fit by the Freundlich isotherm, as evidenced by data linearity in this region. However, as the isotherm reached saturation, the data deviated from the Freundlich isotherm, and this high concentration region was well modeled by the Langmuir isotherm.

## Conclusions

The present study reports the potential of lotus root biomass for the sorption of As (III) from an aqueous sample. The biosorption process was found to be dependent on various process parameters such as pH, agitation speed, contact time, As (III) concentration, and biosorbent dose. Maximum sorption was at pH 7, and equilibrium was achieved in only 60 minutes. Langmuir and Freundlich equilibrium isotherm equations were used to describe experimental adsorption data. Based on the correlation co-efficient, it was observed that the Langmuir isotherm provides the best correlation for experimental data with a coefficient of determination of 0.99. This suggests that adsorption of arsenic was limited with monolayer coverage and the surface was relatively homogenous in terms of active sites. Furthermore, the *R*_*L*_ value in the present investigation was less than one, indicating that the adsorption of the As (III) onto lotus root biomass was favorable. The loading capacity of lotus root biomass for arsenic was found to be 1:20, which is higher compared to other adsorbents studied thus far. Lotus root biomass obtained from lotus root was found to be an effective, inexpensive adsorbent and showed quantitative sorption (96%) of As (III) from aqueous solution. The proposed method can provide an alternative simple, low cost-efficient methodology for arsenic sorption from water/wastewater and is suitable for application in small-scale industries.
